# Disorganization, *COMT*, and Children's Social Behavior: The Norwegian Hypothesis of Legacy of Disorganized Attachment

**DOI:** 10.3389/fpsyg.2016.01013

**Published:** 2016-07-12

**Authors:** Zhi Li, Beate W. Hygen, Keith F. Widaman, Turid S. Berg-Nielsen, Lars Wichstrøm, Jay Belsky

**Affiliations:** ^1^Department of Human Ecology, University of California, DavisDavis, CA, USA; ^2^NTNU Social ScienceTrondheim, Norway; ^3^Department of Psychology, Norwegian University of Science and TechnologyTrondheim, Norway; ^4^Graduate School of Education, University of California, RiversideRiverside, CA, USA; ^5^Regional Center for Child and YouthMental Health and Child Welfare, Norwegian University of Science and TechnologyTrondheim, Norway

**Keywords:** attachment disorganization, *COMT*, social behavior, confirmatory analyses, replication

## Abstract

Why is disorganized attachment associated with punitive-controlling behavior in some, but caregiving-controlling in others? Hygen et al. (2014) proposed that variation in the Catechol-O-methyl transferase(*COMT*) Val158Met genotype explains this variation, providing preliminary data to this effect. We offer a conceptual replication, analyzing data on 560 children (males: 275) drawn from the NICHD Study of Early Child Care and Youth Development. As predicted, competitive model-fitting indicated that disorganized infants carrying Met alleles engage in more positive behavior and less negative behavior than other children at age 5 and 11, with the reverse true of Val/Val homozygotes, seemingly consistent with caregiving-controlling and punitive-controlling styles, respectively, but only in the case of maternal and not teacher reports, thereby confirmating a relationship-specific hypothesis.

## Introduction

In contrast to a secure-attachment history, one of insecure attachment early in life is associated with more problematic functioning later in development, including externalizing problems (e.g., Fearon et al., 2010; Groh et al., 2012) and internalizing ones (e.g., Shaw et al., 1997; Brumariu and Kerns, 2010; Colonnesi et al., 2011). Among children lacking a secure attachment, the most problematic development is usually manifested by those classified as disorganized (e.g., Carlson and Sroufe, 1995; Lyons-Ruth, 1996; Lyons-Ruth et al., 1997; Carlson, 1998; Van Ijzendoorn et al., 1999; Moss and St-Laurent, 2001; Fearon et al., 2010).

Of note, however, is the distinctive variation in how disorganization manifests itself as children develop beyond the infancy years. Thus, investigators have distinguished a controlling-caregiving style from a more controlling-punitive style (see detailed description of the two disorganization styles in Section Types of Preschool Disorganized Attachment). Such distinct patterns are thought to be the result of differential experience with the parent and characteristics of the children themselves, including severity of their disorganization (George and Solomon, 1998; Moss et al., 2004; Solomon and George, 2006; Bureau et al., 2009). In the current inquiry, we consider an alternative explanation of variation in the way children with disorganized attachment histories develop, one recently advanced by a team of mostly Norwegian investigators—which we refer to as the “Norwegian hypothesis” (Hygen et al., 2014). It stipulates that the genetic make-up of the child affects how disorganization manifests itself, highlighting in particular the Catechol-O-methyl transferase (*COMT*) val158Met genotype. We thus conduct a conceptual replication of the gene -X-disorganization interaction central to the Norwegian hypothesis as delineated below, drawing on data from the NICHD Study of Early Child Care and Youth Development (NICHD Early Child Care Research Network, 2005). We regard this effort as especially important in light of concern for the replicability of scientific findings (Jasny et al., 2011; Ryan, 2011). Because we ourselves were skeptical about the predictions to be tested, we planned a restricted set of analyses, 100% of which are presented here, allowing “the empirical chips to fall wherever they may.”

### Types of preschool disorganized attachment

The social behavior of children develops substantially in the period between the end of infancy and the start of school (as well as thereafter). Whereas prosocial behavior increases notably over this time period (Eisenberg and Fabes, 1998; Benenson et al., 2003), physical aggression declines after peaking sometime between 2 and 4 years of age (Tremblay et al., 2004; Côté et al., 2006). Children with disorganized histories deviate from typical developmental trajectories, at least with regard to behavior directed toward their mothers, but seem to do so in two contrasting ways (Main and Cassidy, 1988; Wartner et al., 1994; Moss et al., 2005, 2011; O'connor et al., 2011). Those who display the controlling-punitive style of disorganization behave in ways that are physically threatening (e.g., aggression) and verbally harsh and commanding. It is no wonder, then, that their parents characterize them as aggressive, irritable, hyperactive, unadaptive, confrontational, moody, and hard to control (George and Solomon, 1996). One might expect them, therefore, to manifest especially elevated levels of aggression and lower levels of social skills (e.g., cooperation) on standard measures of social functioning, at least when parents themselves are characterizing child behavior.

Disorganized children judged to be controlling-caregiving exhibit a rather different behavioral profile. They direct the parent's activity and conversational exchanges by structuring interactions in a helpful and/or emotionally positive manner (Main and Cassidy, 1988; Cassidy and Marvin, unpublished manuscript). By being exceptionally cheerful, polite, or supportive, such children seem especially attentive to the moods and needs of parents and motivated to protect them (Solomon and George, 2011). Once again it is not surprising that the parents of these disorganized children perceive them to be especially close, adaptable, and responsible, even socially precocious, taking responsibility for the emotional well-being of their parents. It is also not surprising that parents as well as naïve observers might mistakenly regard the controlling-caregiving behavior of these children as a sign of social competence, cooperation, and positive development. Theory and research suggest, however, that this is a defensive posture, representing a strategy of inflating or maximizing prosocial behavior directed toward the parent in an effort to avoid displeasing the parent and, thereby, engendering conflict. This latter perspective implies that the elevated levels of positive social behavior and low levels of aggressive behavior that one might expect to document when relying on parental reports of these attributes reflect less social competence than overcompensation and a means of self-protection which results from fear of the frightening or frightened parent. There is also the view that the family processes of parentification and role reversal—which involve the child assuming practical and/or emotional responsibility for the parent, including care of siblings and managing the household—are driven by children's need to protect the attachment system from the frequent disruptions in the child-caregiver relationships, disruptions which themselves derive form frightening caregiver behavior and are often associated with impaired child psychological health (e.g., suicidal ideation, self-harm) (Bifulco et al., 2014).

The core issue we address in the research reported herein concerns which kindergarten-age children with disorganized attachments in infancy manifest what would appear to be these divergent styles of behavior, as reflected in standard measures of social functioning. Because our focus is not on the specific parenting that others have used to account for these two different legacies of disorganized attachment, but rather on the child's genotype, we next consider research on the genetics of attachment.

### The genetics of attachment

Behavior-genetic studies of attachment early in life have indicated, repeatedly, that unlike so many other psychological-behavioral characteristics, attachment security is not (apparently) heritable (Bokhorst et al., 2003; Roisman and Fraley, 2008). Notable as well is that molecular-genetic efforts to link candidate genes with measures of attachment early in life have failed to chronicle reliable associations. (Luijk et al., 2011; Mesquita et al., 2013; Roisman et al., 2013). Despite such seemingly consistent evidence, one can still wonder whether genetics plays a role in how the developmental legacy of early attachment—that is, its sequelae—is expressed. Recent research addressed this issue of gene -X-attachment interaction in predicting later social functioning, with four studies indicating that children carrying short alleles in the 5-HTTLPR promoter region with histories of insecure, but not secure attachment, underperform other insecure children carrying long alleles on measures of autonomy (Zimmermann et al., 2009), stress response (Gilissen et al., 2008; Frigerio et al., 2009); and regulatory control (Kochanska et al., 2009). Insecurely attached children also have been observed to respond in more stressful ways than secure infants when carrying the CC allele of the GABRA6 genotype or the Val/Val variant of the *COMT* gene (Frigerio et al., 2009). A fifth study detected a significant interaction between FKBP5 rs1360780 and insecure-resistant attachment in predicting stress reactivity, such that infants with this attachment history carrying one or two T-alleles showed elevated levels of cortisol (relative to baseline) in the Strange Situation in comparison to those lacking T alleles (Luijk et al., 2011).

More recently—and especially important to the current inquiry—Hygen et al. (2014) predicted and documented a significant interaction between disorganization at age 4 years—as measured using a doll-play procedure (i.e., MCAST, Green et al., 2000)—and *COMT*, on *change in social behavior* from age 4–6 years. It is thus a *COMT*-X-disorganization interaction that we seek to conceptually replicate herein. The Norwegian team chose to focus on the *COMT* polymorphism because of suggestive evidence linking it to both disorganization and aggression (Baud et al., 2007; Kulikova et al., 2008; Albaugh et al., 2010), the latter being one of their core outcomes.

Notable as well is that that variation in *COMT* activity influences variation in dopamine levels, particularly in the PFC. In fact, the *COMT* enzyme accounts for more than 60% of dopamine degradation in the PFC (Karoum et al., 1994). Thus, individuals homozygous for the Val allele of *COMT* Met158Val have four times as much *COMT* activity as Met homozygotes, resulting in lower dopamine level in the PFC (Lachman et al., 1996; Weinshilboum et al., 1999). It was also as a result of these observations that the Norwegian team assumed that children homozygous for the Val allele would have lower levels of dopamine than Met-carrying children. This expectation was central to their specific prediction as to how *COMT* would moderate the effects of disorganization on social functioning reflective of controlling-caregiving and controlling-punitive behavioral styles. Also influencing their predictions was evidence that childhood sexual abuse (Perroud et al., 2010), or existing behavior problems, such as ADHD (Caspi et al., 2008), forecast elevated levels of aggression and that *COMT* in combination with ADHD has been linked to impaired social understanding (Langley et al., 2010). Indeed, it was for these reasons that they focused, as we do, too, on aggression and social competence.

What Hygen et al. (2014) predicted—and found—was that whereas 4-year olds who scored high on the doll-play measure of disorganization and were homozygous for Val alleles *increased* in their levels of aggression and *decreased* in social skills across the transition to school, those carrying Met alleles changed in exactly the *opposite* direction (i.e., decreased aggression, increased social skills). Surprisingly, as already implied, the *COMT* X disorganization interaction did not predict, as originally anticipated by the Norwegian investigators, level of functioning at either ages four or six, but predicted only change over time. The latter result did prove relationship specific, as anticipated, however, in that the gene-X-attachment interaction predicted child behavior reported only by mothers, not by teachers.

### Current study

We sought to extend Hygen et al. (2014) research by testing “the Norwegian hypothesis” regarding the differential and genetically moderated developmental legacies of attachment disorganization. Thus, consistent with the Norwegian investigators' original hypothesis, we anticipated that the *COMT* genotype would predict similarly divergent patterns of social functioning in the case of kindergarteners classified as disorganized in their attachments to their mothers at age 15 months, but only when maternal rather than teacher reports of child behavior were subject to analysis; and that the just delineated genetic moderation would not be evident in the case of children with non-disorganized attachment histories. We chose to focus on level of functioning at kindergarten age rather than change in behavior over time (as the Norwegian investigators did), because this is where the Norwegian investigators initially expected to chronicle *COMT*-moderated effects of disorganized attachment. Notably, instead of relying on a doll-play measure of disorganization at age 4 years, we examine disorganization assessed in the gold-standard Strange Situation at age 15 months. And instead of focusing upon change in behavior from age 4 to 6 years, we focus on behavioral functioning in kindergarten, some 3.5 years following assessment of early attachment. Furthermore, in secondary analyses, we evaluate the *COMT*-X- disorganization interactions on the same (mother- and teacher-reported) child behavior measures in grade six to determine whether any interaction detected at an earlier age proves evident years later. Because of these method and design distinctions, we regard the current inquiry as a conceptual rather than exact replication.

In contrast to “exact replications,” one can adopt less stringent criteria and still speak of replication. A “conceptual replication,” like the one being reported herein, may prove somewhat different from what was done in prior work, but is yet *conceptually* parallel to and informed by the work for which “replication” is sought. The essential question becomes not whether specific findings tied to specific measurements evaluated in a seemingly identical sample can be repeated, but whether the more general finding can be detected again using related, even if not exactly the same, measurements or research design. Indeed, this is more or less why meta-analyses are undertaken—to determine whether, across often diverse studies using different methods and samples, some finding emerges with some degree of reliability, as well as what investigatory factors might affect whether or not a finding emerges. In the research reported herein, we were motivated to conduct what should be regarded as a conceptual replication.

Importantly, instead of conducting a traditional—and exploratory—analysis to determine whether the statistical interaction of disorganization and *COMT* predicts parent- and/or teacher-reported measures of aggression and social competence in kindergarten, we employ a confirmatory and competitive model-fitting strategy. We adopted this statistical approach because the current work attempts to replicate conceptually the findings of the Norway group, being based as it is on explicit, a-priori predictions about how the data will be *patterned* (see next paragraph). Thus, model fitting depends on clearly formulated propositions as to how the data will be organized, with the overall fit of the model being most important, rather than one or another particular component of the model. This approach contrasts markedly, then, to an exploratory regression where one may even anticipate a significant interaction but makes no commitment to what exact form it will take, thereby requiring follow-up or decomposition tests to illuminate the form of the interaction (Belsky et al., 2013).

**Figure 1:**
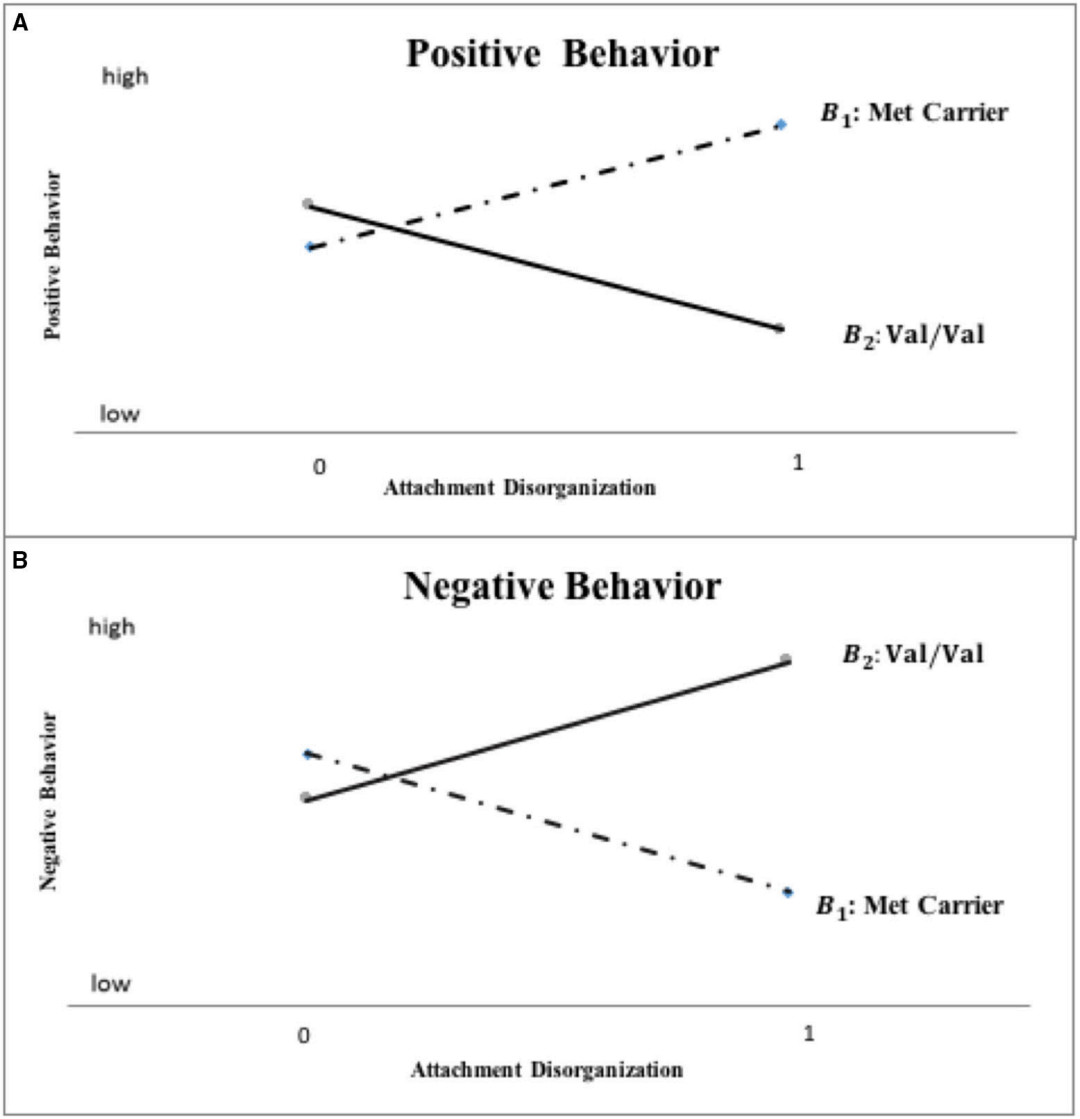
**Hypothesized *COMT* X disorganization interaction pattern for mother-reported positive and negative child behavior (“0” = organized; “1” = disorganized)**.

Based on the original Norwegian hypothesis, this conceptual replication specifically, directly and *collectively* tests the following multiple propositions graphically depicted in Figure 1: (a) kindergarteners with a history of disorganized attachment who are homozygous for the Val allele will score higher on aggression and lower on social competence, reflecting a punitive-controlling behavioral style, than will non-disorganized/organized children; (b) kindergarteners with a history of disorganized attachment carrying the Met allele, will score lower on aggression and higher on social competence, reflecting a caregiving-controlling behavioral style than will non-disorganized/organized children; and (c) because disorganization, by definition, refers to the nature of a relationship that a child has with his or her caregiver, and that children's punitive and caregiving behavior are only activated by, and within the (parent-child) attachment system, these differential developmental legacies of disorganized attachment will be evident only in maternal, not teacher reports; finally (d) children who do not have a history of disorganized attachment will not exhibit differential aggression or social competence outcomes as a function of their *COMT* gene allelic status.

## Material and methods

### Participants

Participants were recruited during the first 11 months of 1991 in 24 hospitals from 10 data- collection locations in the United States (Charlottesville, VA; Irvine, CA; Lawrence, KS; Little Rock, AR; Madison, WI; Morganton, NC; Philadelphia, PA; Pittsburgh, PA; Seattle, WA; and Wellesley, MA). A total of 8986 women who gave birth in selected 24-h intervals were screened for eligibility, and 1364 families with healthy newborns completed a home interview when the infant was 1 month and ultimately became participants. The study was reviewed and approved by University of Wisconsin Internal Review Board. More details about recruitment and selection procedure can be found in NICHD Early Child Care Research Network (2005).

In the current study, only children of Caucasian ethnicity were included to avoid confounding ethnicity and gene frequency. Among the 693 children whose genetic information was available, 579 were Caucasian, and 19 of these children were excluded from the analysis sample due to the missing information on attachment security. The final sample for the model-fitting analyses consists of 560 children (Boys: 275; Girls: 285).

### Measurements

#### Attachment disorganization at 15 months

Attachment security was measured at 15 months using the Ainsworth and Wittig (1969) Strange Situation Procedure (SSP). Infant attachment was classified, based on careful review of videotapes, into one of four major categories: secure (B), avoidant (A), resistant (C) or disorganized (D). Disorganized (D) infants do not display a coherent strategy in the SSP. For example, they may exhibit a mixture of avoidant and resistant behavior or seem very confused or apprehensive upon reunion with mother. Cases that cannot be categorized are designated as unclassifiable (U) or missing. All SSP videotaped were later shipped to a central location for coding. 80 children were categorized as avoidant (A, 14.3%); 336 were assigned to the secure category (B, 60%); 49 children were coded as resistant (C, 8.75%); 72 children were classified as disorganized (D, 12.9%), and 23 children proved unclassifiable or were missing attachment data (21 U, 3.75% and 2 with missing code, 0.4%). In this effort to conceptually replicate the findings of the Norwegian study, all non-disorganized classifications were combined to create a non-D group (*N* = 488)—to be compared with those classified disorganized (*N* = 72)—in the primary analyses; in secondary, sensitivity analyses, the continuous D rating was used. Each SSP tape was coded by two coders working independently. Across all coder pairs, agreement with the five-category classification system was 83% (kappa = 0.69) [NICHD (Early Child Care Research Network), 1997].

#### COMT

Buccal mucosa cells were collected with cotton swabs by the subjects at 15 years of age. DNA extraction and genotyping for the NICHD Study of Early Child Care and Youth Development (SECCYD) were performed at the Genome Core Facility in the Huck Institutes for Life Sciences at Penn State University under the direction of Deborah S. Grove, Director for Genetic Analysis. Taqman SNP Genotyping Assays were performed using an Allelic Discrimination Assay (Applied Biosystems, Foster City, CA) protocol. Forty nanograms of DNA were combined in a volume of 5 microliters with 2X Universal PCR Mix (Applied Biosystems) and 1/20 the volume of the Taqman SNP assay in a 384 well plate. A Pre-Read was performed and then PCR as follows: a 10 min hold at 95°C, followed by 40 to 45 cycles of 15 s at 92°C and then 1 min at 60°C in a 7900HT PCR System. After amplification, a Post-Read was performed, and automatic and manual calls were made. The genotypes of the participants were categorized as Val/Val (GG, *N* = 158, 27.3%), Val/Met (AG, *N* = 272, 47.0%) and Met/Met (AA, *N* = 149, 25.7%). The distribution of the genotypes did not deviate from Hardy-Weinberg equilibrium (χ^2^ = 2.10, *p* > 0.05). Following the Norwegian study we are attempting to conceptually replicate, all Met carriers (*N* = 421), whether Val/Met (AG) heterozygotes or Met/Met (AA) homozygotes formed one genotypic subgroup initially—in the primary analyses (see Data Analysis Plan below)—to be compared with the group of Val/Val (GG) homozygotes (*N* = 158). In secondary, sensitivity analyses, subgroup scoring was modified. Collectively, then, there were (a) 353 organized Met carriers, (b) 135 organized Val/Val homozygotes, (c) 55 disorganized Met carriers and (d) 17 disorganized Val/Val homozygotes. Chi-square tests indicated no association between the *COMT* genotype (Met carriers vs. Val/Val) and children's disorganization classification (D vs. Not-D) [χ^2^(1) = 0.52, *p* = 0.47]. The independence between *COMT* genotype and children's attachment classification—regarded as a proxy for the environment— indirectly ruled out the possibility of passive gene-and-environment associations(rGE). Thus, the Disorganization-X-*COMT* interaction revealed in this study could not be attributed to the possibility that disorganized children inherited the *COMT* gene from their parents who then, in response, treated their children problematically, thus resulting in attachment disorganization.

#### Aggressive behavior: mother-report and teacher-report

Aggressive behavior was measured using the Child Behavior Checklist (CBCL) and the Teacher Report Form (TRF) completed by mothers and teachers, respectively, when children were in kindergarten. A list of 118 (CBCL)/120 (TRF) items that includes a broad range of children's behavioral or emotional problems was presented. For each item, the respondent was asked to determine how well that item described the target child currently or within the last 2 months: 0 = Not True (as far as I know), 1 = Somewhat or Sometimes True, and 2 = Very True or Often True. The aggressive behavior subscale score is the sum of 20 mother-report and 25 teacher-report items, resulting in a range of scores of 0–40 for mothers and 0–50 for teachers. A higher score indicates greater aggressive behaviors. (Descriptive information was presented in Table 1).

**Table 1:** **Sample characteristics before multiple imputation**.

	**Entire sample (*N* = 560)**			**Disorganized group (*N* = 72)**			**Non-disorganized group (*N* = 488)**		
	***N***	**Mean (SD)**	**[Min, Max]**	***N***	**Mean (SD)**	**[Min, Max]**	***N***	**Mean (SD)**	**[Min, Max]**
**MOTHER-REPORT**									
Aggression	532	7.55 (5.42)	[0, 26]	68	7.34 (6.15)	[0, 26]	464	7.59 (5.32)	[0, 26]
Cooperation	531	12.88 (2.99)	[4, 19]	68	13.28 (3.29)	[5, 19]	463	12.82 (2.94)	[4, 19]
Assertion	531	17.22 (2.27)	[9, 20]	68	17.28 (2.03)	[11, 20]	463	17.21 (2.30)	[9, 20]
Responsibility	531	13.20 (2.79)	[5, 20]	68	13.84 (2.51)	[7, 18]	463	13.11 (2.82)	[5, 20]
Self-control	531	12.65 (3.13)	[3, 20]	68	12.94 (3.32)	[6, 20]	463	12.61 (3.11)	[3, 20]
**TEACHER-REPORT**									
Aggression	509	3.89 (6.53)	[0, 40]	66	5.52 (9.19)	[0, 40]	443	3.64 (6.01)	[0, 38]
Cooperation	503	16.51 (3.55)	[2, 20]	66	16.74 (3.63)	[4, 20]	437	16.47 (3.54)	[2, 20]
Assertion	503	13.28 (4.08)	[1, 20]	66	13.56 (3.89)	[5, 20]	437	13.24 (4.11)	[1, 20]
Self-control	503	15.51 (3.54)	[3, 20]	66	15.85 (3.78)	[3, 20]	437	15.46 (3.50)	[4, 20]

SD, standard deviation; Min, minimum value; Max: maximum value.

#### Social competence: mother-report and teacher-report

Social competence was assessed by 38-item mother-report and 30-item teacher-report form of the Social Skills Rating System (SSRS) (Gresham and Elliott, 1990). Three subscale scores derived from both of these (maternal- and teacher-report) instruments are Cooperation (coefficients α = 0.76 (mother)/0.92 (teacher); e.g., “volunteer to help family members”), Assertion (αs = 0.69 (mother)/0.86 (teacher); e.g., “make friends easily”), and Self-control (αs = 0.80(mother)/0.87(teacher); e.g., “control temper when arguing with other”). The longer, mother-report instrument also includes a subscale for Responsibility (α = 0.62(mother); e.g., request permission before leaving house). (Descriptive information was displayed in Table 1).

### Data analysis plan

Data analyses proceeded in three major phases: preliminary, primary, and secondary. Preliminary analyses involved multiple imputation of missing data (which ranged from 5.0 to 9.1% across dependent variables) (Rubin, 1987; Schafer, 1997; Schafer and Graham, 2002) using the Markov Chain Monte Carlo (MCMC) imputation method to generate 100 imputed data sets for all cases on whom *COMT* and disorganization data were available. Primary analyses testing the core hypothesis central to this inquiry were thus run 100 times, once using each imputed data set, with results composited across these multiple analyses. More specifically, using SAS PROC MIANALYZE, parameter estimates were averaged across imputed datasets, with the corresponding standard errors of the parameter estimates calculated after accounting for the variances from sampling and multiple imputation, according to Rubin's (1987) rule.

The second step of preliminary analysis, the results of which are reported now, focused on data reduction. More specifically, exploratory factor analyses were used to determine whether the measures of child functioning could be composited based on empirical grounds, thereby reducing the number of statistical tests conducted and the likelihood of generating chance results. Two rather clear factors emerged which afforded creation of two composite measures, one of positive and one of negative behavior. Eigenvalue of both factors were greater than one. The first (positive-behavior) factor explained 76% and the second (negative-behavior) factor explained 24% of variance. Factor loadings of variables from the first factor used to create the positive-behavior composite ranged from 0.46 to 0.91 and those from the second factor used to create the negative-behavior composite ranged from 0.58 to 0.95. There were no cross-loaded items within these ranges on either factor. Thus, a *Positive Behavior* score was created for mothers by summing standardized scores for cooperation, assertion, and responsibility, though in the case of teachers, only the first two subscales were composited (as there was no score for responsibility). A *Negative Behavior* score was created by summing standardized scores for aggression and self–control for both maternal and teacher reports of child behavior, with the latter variable reversed to reflect lack of self-control.

The primary analyses tested hypotheses central to this inquiry using a confirmatory, model-testing approach with genetic subgroups scored as in the original Norwegian research and with respect to the kindergarten outcome measures. This model-fitting approach allows for direct evaluation of the overall *pattern* of the data and whether it proves consistent with the multiple hypotheses outlined at the end of the Introduction. Specifically, the approach assumes that the difference on outcome variables between the Met carrier and Val homozygote groups will be significant only for children with disorganized attachment, only in the case of parent reports and, based on prior work, this difference should take a specific form. That is, the difference between Met carriers and Val homozygotes on mother-reported outcomes will be significant only for children with disorganized attachment, with Val homozygotes exhibiting lower mother-reported positive-behavior and higher negative-behavior scores than Met carriers. In contrast, children with non-disorganized attachment (i.e., the secure, insecure-avoidant, and insecure-resistant attachment groups) should not exhibit different outcomes as a function of their genetic status in the case of either mother- or teacher-reported outcomes (see Figure 1).

To instantiate these predictions, the statistical model stipulates that:

(1) $$\{\begin{array}{c} group\;=\;1:Y\;=\;A_{1}\;+\;B_{1}X\;+\;E\\ group\;=\;2:Y\;=\;A_{2}\;+\;B_{2}X\;+\;E \end{array}$$

In this model all parameters were freely estimated: *A*_1_ and *A*_2_ are the intercept for each genetic subgroup (group = 1: Met Carrier (i.e., Val/Met and Met/Met); group = 2: Val/Val); *B*_1_ and *B*_2_ represent the differential slope for each genetic subgroup; and E refers to the error term. *A*_1_ should not differ from *A*_2_, but *B*_1_ should differ—*and in the hypothesized direction*—from *B*_2_. The difference between *A*_1_ and *A*_2_ is, essentially, the simple main effect of genetic subgroup (Met vs. Val) for children with non-disorganized attachment, and we predict that the Met and Val subgroups will not differ for children with non-disorganized attachment. Furthermore, the test of the difference between *B*_1_ and *B*_2_ is a test of the genetic subgroup (Met carriers vs. Val homozygotes) X attachment group (nondisorganized vs. disorganized), and we predict that children with disorganized attachment will differ significantly from their counterparts with nondisorganized attachment as a function of their genetic make-up—*and in the hypothesized direction*. Notably, the most crucial tests of our theoretical predictions are the tests of difference between *B*_1_ and *B*_2_, reflecting the differences of disorganized Met carriers and Val/Val individuals from their genetic counterparts with nondisorganized attachment. This test, together with the model comparison described below, allowed us to assess the overall patterning of the data. Importantly, although our theoretical approach specified the direction of the differences (e.g., differences between *B*_1_ and *B*_2_), the significance of the simple slopes (e.g., *B*_1_) was *not* the primary prediction and thus is not the focus of the current analyses.

Based on the equation shown above, the test of difference between *B*_1_ and *B*_2_ is a test of the gene-X-disorganization interaction, because statistical significance of this test is an indicator that the regression lines differ as a function of genetic subgroup, hence the presence of an interaction. The equation shown above is a four-parameter equation, with parameters of *A*_1_ and *A*_2_ and *B*_1_ and *B*_2_; we termed this model the *full* model. To evaluate whether the difference between *A*_1_ and *A*_2_ was negligible, we formulated a nested three-parameter equation constraining *A*_1_ = *A*_2_, which we term our *hypothesized* model, because the difference between non-disorganized Met carriers and Val homozygotes is nil, but the difference between disorganized Met carriers and Val homozygotes, reflected in the *B*_1_ and *B*_2_ parameters, remains in the model. We also fit another three-parameter model, termed the *comparison* model, in which we constrained *B*_1_ = *B*_2_, but allowing *A*_1_ and *A*_2_ to be separately estimated. Thus, the hypothesized model posits that the genetic subgroups will not differ for children with non-disorganized attachment, but they will differ for those with disorganized attachment. Conversely, the comparison model assumes that the genetic subgroups may differ for children with non-disorganized attachment, but will not differ at all for those with disorganized attachment.

We competitively evaluated the relative fit of these three models with the Bayesian information criterion (BIC), which can be used to evaluate the fit of non-nested models, such as the *hypothesized* and *comparison* three-parameter models. Lower values of the BIC indicate better fit of a model to data. We predicted that the *hypothesized* model would yield better fit to data than both the *full* model and the *comparison* model for mother-report outcomes, but not for teacher-report outcomes. The primary analyses also evaluated the effect sizes of the *COMT-*X-disorganization interactions on kindergarten mother-reported positive and negative behavior by magnitude of differences (i.e., Cohen's d. http://www.campbellcollaboration.org/resources/effect_size_input.php).

The secondary analyses involved evaluating the sensitivity of the results which emerge from the primary analyses using (1) outcomes measured at Grade 6 and (2) scoring genetic subgroups and disorganization differently than in the primary analyses when again predicting kindergarten and Grade-6 outcomes. In the latter analyses, we left the *COMT* coding in its original form, where groups 1, 2, and 3 represented persons with 0, 1, or 2 Val alleles, respectively, and we left the disorganization rating in its full 9-point format, coded 0 = no evidence of disorganization to 8 = highly disorganized. We then fit the following model:

(2) $$\{\begin{array}{c} group\;=\;1:Y\;=\;A_{1}\;+\;B_{1}X\;+\;E\\ group\;=\;2:Y\;=\;A_{2}\;+\;B_{2}X\;+\;E\\ group\;=\;3:Y\;=\;A_{3}\;+\;B_{3}X\;+\;E \end{array}$$

The preceding equation is similar to Equation 1, but has slope and intercept estimates for each of the *COMT* allele groups. The model above has 6 parameter estimates—three intercepts (A1 through A3) and three slopes (B1 through B3). We also fit a linear *COMT* X linear disorganization model as a 4-parameter model, in which we constrained the intercept for group 2 to fall exactly between the intercepts for groups 1 and 3, and a similar constraint was places on the slope values. Because the 4-parameter linear X linear model always had a lower BIC value, and therefore better model fit, relative to the 6-parameter full model, we report only results for the linear X linear model below.

## Results

### Primary analyses

**Figure 2:**
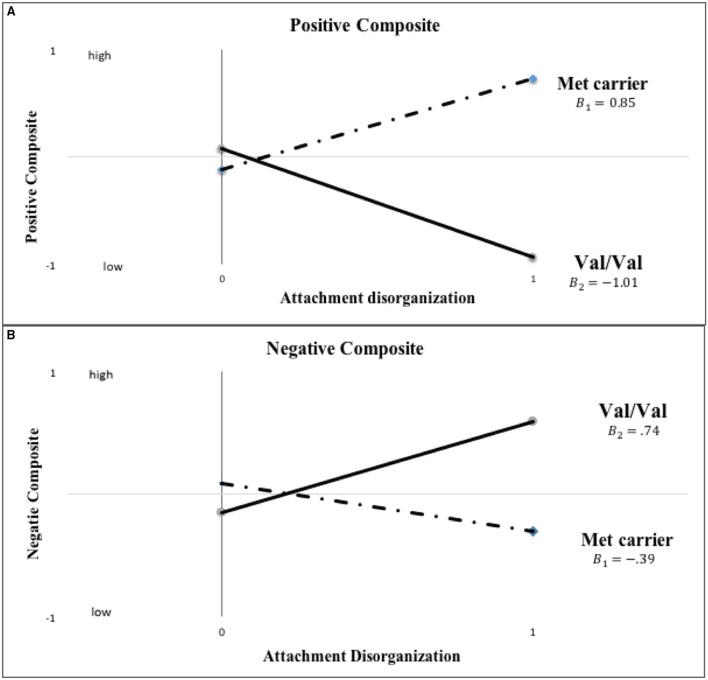
**Actual *COMT* X disorganization interaction pattern for *kindergarten* mother-reported positive and negative child behavior (“0” = organized; “1” = disorganized)**. Note: The primary analyses used binary *COMT* coding (i.e., Val/Val vs. Met carrier) and categorical disorganization score (“0” = organized; “1” = disorganized).

Detailed results of the primary data analyses are displayed in Table 2, but before highlighting them, it is important to consider BIC values and thus the overall fit of the multiple models tested, as these most directly address the overall patterning of the data. Comparisons of the full, hypothesized, and comparison models using the BIC statistic in the case of mother-reported child behavior showed that the hypothesized model fit the data best. For each of the 100 imputed data sets, the hypothesized model had a lower (i.e., better) BIC value than the full model on the mother-rated positive composite (BIC difference: *M* = −5.57, *SD* = 0.36) and negative composite (BIC difference: *M* = −4.51, *SD* = 0.46). In addition, for each imputed data set, the hypothesized model had a lower, better BIC value than the comparison model (positive behavior BIC difference: *M* = −6.20, *SD* = 0.65; negative behavior BIC difference: *M* = −2.73, *SD* = 0.52). More specifically and as graphically displayed in Figure 2, disorganized Met carriers(β_*Met*−*carriers*_ = 0.85, *p* = 0.02) scored significantly *higher* on mother-reported positive behavior than disorganized Val/Val homozygotes(β_*Val*∕*Val*_ = −1.01, *p* = 0.10) (*d* = 0.722; 95% CI: [0.165, 1.278]), but *lower*, even if not to a significant extent, in the case of negative behavior (i.e., Val/Val > Met carriers; *d*= −0.448; 95% CI: [-0.997, 0.101]; β_*Met*−*carriers*_ = −0.39, *p* = 0.11; β_*Val*∕*Val*_ = 0.74, *p* = 0.11).

**Table 2:** **Confirmatory analyses of the effects of Attachment Disorganization, *COMT* and their interaction on the mother- and teacher-report ratings when children were in kindergarten (*N* = 560)**.

	**Positive composite**		**Negative composite**	
**Parameter**	**Estimate (*SE*)**	***t* (*p*)**	**Estimate (*SE*)**	***t* (*p*)**
**MOTHER-REPORTS**				
*A*_1_	−0.12 (0.13)	−0.92 (0.38)	0.09 (0.10)	0.93 (0.36)
*B*_1_	0.85 (0.36)	2.39 (0.02)^*^	−0.39 (0.27)	−1.46 (0.11)
*A*_2_	0.08 (0.21)	0.40 (0.69)	−0.15 (0.16)	−0.97 (0.33)
*B*_2_	−1.01 (0.62)	−1.63 (0.10)	0.74 (0.46)	1.59 (0.11)
*A*1 = *A*_2_	−0.20 (0.25)	−0.83 (0.41)	0.24 (0.18)	1.31 (0.19)
*B*1 = *B*_2_	1.86 (0.71)	2.61 (0.009)^**^	−1.13 (0.53)	−2.11 (0.03)^*^
BIC full model	2594.23 (7.19)		2271.54 (8.91)	
BIC hypothesized model	2588.65 (7.14)		2267.02 (9.01)	
BIC comparison model	2594.86 (6.92)		2269.76 (8.75)	
**TEACHER-REPORTS**				
*A*_1_	0.001 (0.09)	0.01 (0.99)	−0.06 (0.10)	−0.60 (0.55)
*B*_1_	0.13 (0.26)	0.48 (0.62)	0.19 (0.28)	0.69 (0.49)
*A*_2_	−0.03 (0.15)	−0.17 (0.87)	0.02 (0.16)	0.10 (0.92)
*B*_2_	0.12 (0.44)	0.28 (0.78)	0.21 (0.47)	0.45 (0.65)
*A*1 = *A*_2_	0.03 (0.18)	0.15 (0.88)	−0.08 (0.19)	−0.40 (0.69)
*B*_1_ = *B*_2_	0.002 (0.51)	0.00 (0.99)	−0.02 (0.55)	−0.03 (0.97)
BIC full model	2202.77 (11.69)		2286.77 (12.42)	
BIC hypothesized model	2196.57 (11.70)		2080.73 (12.40)	
BIC comparison model	2196.47 (11.70)		2080.48 (12.43)	

Tabled values are as follows: Estimate (SE) = point estimate of the parameter, with its associated standard error in parentheses; t (p) = t value for the point estimate, with the p-level of the t statistic in parentheses. The four rows for A_1_, B_1_, A_2_, and B_2_ refer to parameters in Equation 1 based on results from the full model (A_1_and B_1_, intercept and slope for the Met carriers, respectively; A_2_and B_2_, intercept and slope for the Val/Val homozygotes, respectively). The row labeled A_1_ = A_2_ reports results of a test that A_1_ is equal to A_2_, consistent with the hypothesized model; and the row labeled B_1_ = B_2_ reports results of a test that B_1_ is equal to B_2_, consistent with the comparison model. The BIC values reported for the three alternative models are the mean BIC for the model, with SD of BIC values across the 100 imputation samples in parentheses.

* p < 0.05,

** p < 0.01.

In contrast to the mother-reported data, although both the hypothesized and comparison models had smaller BIC values than the full model on the teacher-rated positive- and negative-behavior composites, the hypothesized and comparison models had BIC values that differed very little on these teacher-rated composites (BIC difference positive composite: *M* = −0.10, *SD* = 0.15; BIC difference negative composite: *M* = −0.24, *SD* = 0.30). This indicated that neither model was superior to the other for these teacher-rated composites. As predicted, then, only in the case of maternal-reported child behavior, did the hypothesized model fit the data best.

The statistical foundations of the overall model-fit results just reported are displayed in Table 2 and will now be considered. Note first that the *B*_1_ = *B*_2_ test achieved statistical significance, reflecting the fact that in the case of both *mother*-reported positive and negative composites the genetic groups differed in the expected direction for those with histories of disorganized attachment. The fact that the *A*_1_=*A*_2_ test was not significant indicates that the same difference between genetic subgroups did not emerge for *mother*-reported positive and negative composites in the case of children with organized attachment histories. (i.e., comparing organized Met-carrier with Val/Val individuals, for mother-rated positive composite: Cohen's *d* = −0.082, 95% CI: [−0.282, 0.115]; and for Negative composite: *d* = 0.135; 95% CI: [−0.063, 0.334]. Finally, no differences in either *A* or in *B* parameters emerged in the case of teacher reports.

In sum, even though not every anticipated difference proved statistically significant in its own right, the *patterning* of the entire corpus of data was exactly as predicted, with differences—*in the expected direction*—between genetic subgroups emerging only in the case of maternally reported data, not teacher-reported data, and only in the case of children with a history of disorganization, not children with organized attachment histories. Considered together, then, the gene -X-attachment patterns evaluated on an a-priori basis in the model-fitting analyses appear in line with the proposition that disorganized attachment predicts controlling-caregiving—that is overly nice—behavior in the case of Met carriers, but controlling-punitive—that is, overly hostile—behavior in the case of Val homozygotes. The fact that these differential patterns of behavior were not discerned in the case of teacher-reported outcomes suggests that the discerned results reflect relationship-specific patterns of functioning.

### Secondary analyses

The final set of analyses sought to evaluate the robustness of the findings just reported by means of a series of sensitivity analyses. Thus, to determine whether the *COMT*-X-disorganization findings already reported endured over time the model-fitting analyses just reported were rerun using the same parent- and teacher-reported outcomes measured in 6th grade. We also analyzed parent- and teacher-reported kindergarten and grade 6 positive and negative behavior composites when (a) *COMT* was parameterized as a 3-level variable (0 = Met/Met, 1 = Met/Val, 2 = Val/Val) rather than the original 2-level variable as in the original Norwegian research (i.e., 0 = Met/Met and Met/Val vs. 1 = Val/Val) and (b) using the continuous D (or disorganization) rating from the Strange Situation (*N* = 558) rather than the categorical D classification when testing the predictions central to this inquiry. Because of the three-level coding of *COMT* in multiple robustness checks (i.e., kindergarten, 6th grade), we fitted two sub-models in line with the previous model; the first of these restrained the Met/Val subgroup parameter estimates to fall exactly in between those for the Val/Val and Met/Met subgroups so the gene effect would be constrained to linearity, and the second afforded non-linear genetic effects by freely estimating the slope of Met/Val group.

**Figure 3:**
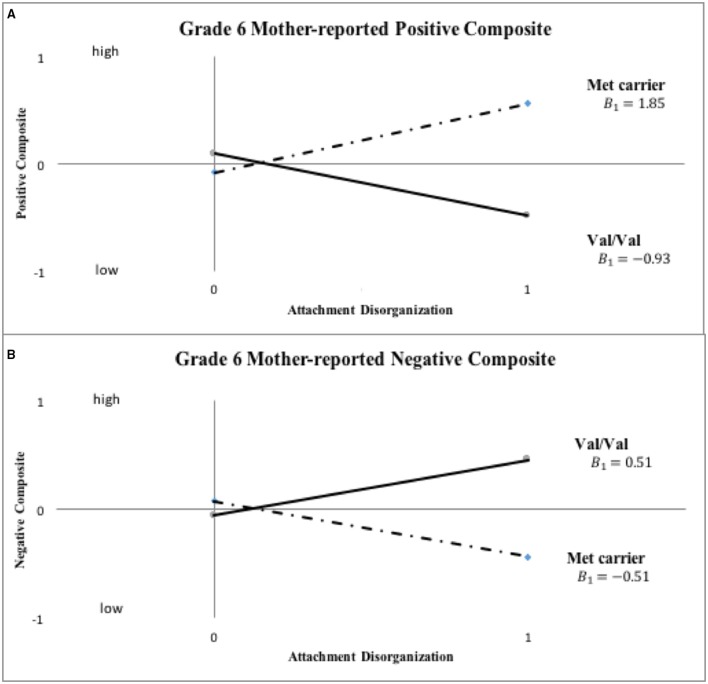
**Sensitivity analyses: *COMT* X disorganization interaction pattern for *Grade 6* mother-reported positive and negative child behavior (“0” = organized; “1” = disorganized)**. Note: This set of sensitivity analyses used binary *COMT* coding (i.e., Val/Val vs. Met carrier) and categorical disorganization score (“0” = organized; “1” = disorganized) to predict grade six child functioning.

**Figure 4:**
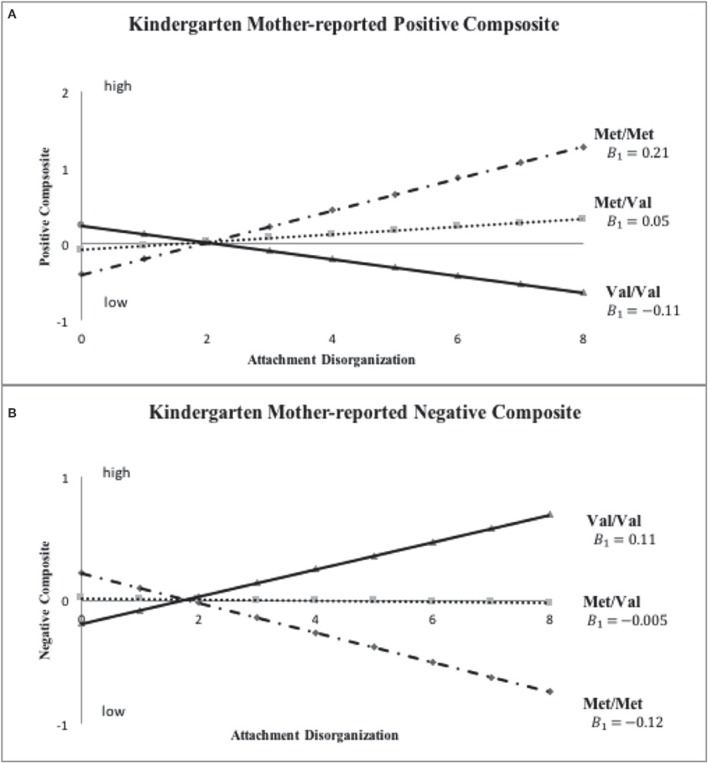
**Sensitivity analyses: *COMT* X disorganization interaction pattern for *Kindergarten* mother-reported positive and negative child behavior**. Note: This set of sensitivity analyses used ternary *COMT* coding (i.e., Val/Val, Met/Val and Met/Met) and continuous disorganization ratings (“0” = organized, “8” = most disorganized).

**Figure 5:**
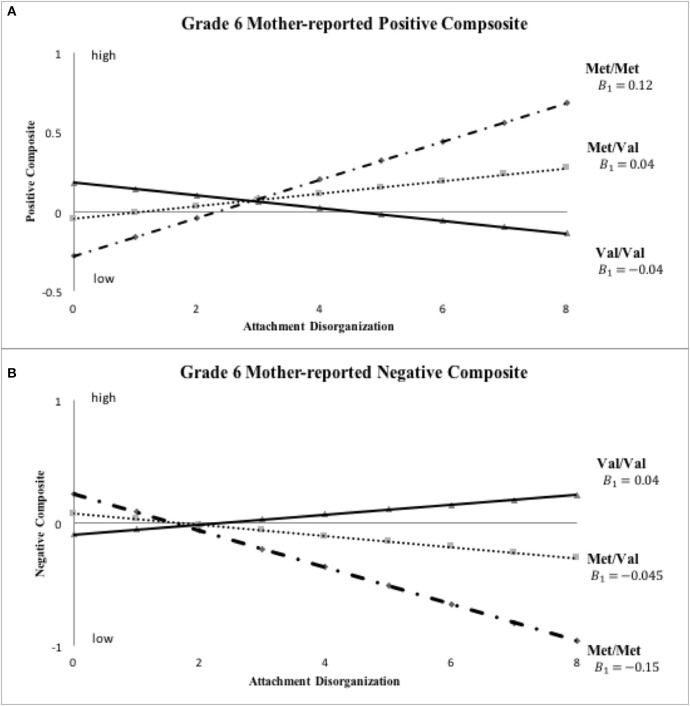
**Sensitivity analyses: *COMT* X disorganization interaction pattern for *Grade six* mother-reported positive and negative child behavior**. Note: This set of sensitivity analyses used ternary *COMT* coding (i.e., Val/Val, Met/Val and Met/Met) and continuous disorganization ratings (“0” = organized, “8” = most disorganized).

Results of these sensitivity analyses, which are presented in Table 3 (i.e., primary analysis re-run at Grade 6) and in Table 4 (i.e., primary analysis re-run using 3-level *COMT* and continuous D at both kindergarten and grade 6), proved *generally* in line with those reported already (see Figures 3–**5**). Before reporting these results in more detail, it should be noted that, as with the original analyses of mother-reported kindergarten outcomes, BIC values proved consistently smaller in the case of the hypothesized model, with the data once again being patterned in a manner in line with the Norwegian hypothesis. For example, in the grade 6 models reported in Table 3, the hypothesized model fit better than the full model in all cases, with ΔBIC values ranging from −4.94 (*SD* = 0.97) to −5.86 (*SD* = 0.16). For kindergarten and grade 6 analyses in Table 4, the linear model fit better than the full model in all cases, with ΔBIC values ranging from −10.74 (*SD* = 0.43) to −12.50 (*SD* = 0.08). Thus, BIC values always supported retention of our hypothesized, constrained models in comparison to more highly parameterized full models.

**Table 3:** **Sensitivity analyses of the effects of Attachment Disorganization, *COMT* and their interaction on the mother- and teacher-report ratings when children were in grade 6 (*N* = 560)**.

	**Positive composite**		**Negative composite**	
**Parameter**	**Estimate (*SE*)**	***t* (*p*)**	**Estimate (*SE*)**	***t* (*p*)**
**MOTHER-REPORTS**				
*A*_1_	−0.08 (0.13)	−0.59 (0.55)	0.07 (0.09)	0.75 (0.45)
*B*_1_	0.64 (0.35)	1.85 (0.06)	−0.51 (0.25)	−2.02 (0.04)^*^
*A*_2_	0.09 (0.21)	0.42 (0.68)	−0.05 (0.15)	−0.38 (0.70)
*B*_2_	−0.58 (0.62)	−0.93 (0.35)	0.51 (0.46)	1.12 (0.26)
*A*1 = *A*_2_	−0.16 (0.24)	−0.67 (0.51)	0.13 (0.18)	0.72 (0.47)
*B*1 = *B*_2_	1.23 (0.71)	1.72 (0.09)^#^	−1.03 (0.52)	−1.96 (0.049)^*^
BIC full model	2589.79 (3.70)		2239.37 (5.95)	
BIC hypothesized model	2583.93 (3.69)		2233.57 (5.95)	
BIC comparison model	2586.54 (3.83)		2237.06 (6.07)	
**TEACHER-REPORTS**				
*A*_1_	−0.08 (0.10)	−0.86 (0.39)	0.02 (0.10)	−0.24 (0.81)
*B*_1_	0.30 (0.26)	1.17 (0.24)	0.14 (0.28)	0.49 (0.62)
*A*_2_	0.11 (0.16)	0.66 (0.51)	−0.13 (0.18)	−0.72 (0.47)
*B*_2_	−0.19 (0.46)	−0.41 (0.69)	−0.10 (0.51)	−0.20 (0.85)
*A*1 = *A*_2_	0.19 (0.19)	1.01 (0.31)	0.15 (0.20)	0.74 (0.46)
*B*_1_ = *B*_2_	0.50 (0.53)	0.93 (0.35)	0.23 (0.57)	0.41 (0.68)
BIC full model	2211.57 (13.06)		2291.83 (14.34)	
BIC hypothesized model	2206.63 (13.04)		2286.38 (14.47)	
BIC comparison model	2206.36 (13.00)		2285.80 (14.38)	

Tabled values are as follows: Estimate (SE) = point estimate of the parameter, with its associated standard error in parentheses; t (p) = t value for the point estimate, with the p-level of the t statistic in parentheses. The four rows for A_1_, B_1_, A_2_, and B_2_ refer to parameters in Equation 1 based on results from the full model (A_1_and B_1_, intercept and slope for the Met carriers, respectively; A_2_and B_2_, intercept and slope for the Val/Val homozygotes, respectively).The row labeled A_1_ = A_2_ reports results of a test that A_1_ is equal to A_2_, consistent with the hypothesized model; and the row labeled B_1_ = B_2_ reports results of a test that B_1_ is equal to B_2_, consistent with the comparison model. The BIC values reported for the three alternative models are the mean BIC for the model, with SD of BIC values across the 100 imputation samples in parentheses.

# p < 0.10,

* p < 0.05.

**Table 4:** **Results of Sensitivity Analysis of Effects of the Attachment Disorganization by *COMT* gene interaction on the parent and teacher-report child outcomes in kindergarten and Grade 6(*COMT*: 3- level coding; Disorganization measured continuously) *(N* = *558)***.

	**Positive composite**				**Negative composite**			
**Parameter**	**Mother-reports**		**Teacher-reports**		**Mother-reports**		**Teacher-reports**	
	**Estimate (*SE*)**	***t* (*p*)**	**Estimate (*SE*)**	***t* (*p*)**	**Estimate (*SE*)**	***t* (*p*)**	**Estimate (*SE*)**	***t* (*p*)**
**KINDERGARTEN**								
*A*1	−0.40 (0.21)	−1.89 (0.06)^#^	0.08 (0.15)	0.53 (0.59)	0.22 (0.16)	1.41 (0.16)	−0.19 (0.16)	−1.18 (0.24)
*B*1	0.21 (0.10)	2.08 (0.04)^*^	0.06 (0.07)	0.08 (0.43)	−0.12 (0.07)	−1.64 (0.10)^#^	−0.004 (0.08)	−0.06 (0.96)
*A*3	0.24 (0.21)	1.14 (0.26)	−0.13 (0.15)	−0.87 (0.38)	−0.19 (0.15)	−1.23 (0.22)	0.09 (0.16)	0.53 (0.60)
*B*3	−0.11 (0.09)	−1.21 (0.23)	−0.003 (0.06)	−0.05 (0.96)	0.11 (0.07)	1.58 (0.12)	0.05 (0.07)	0.78 (0.43)
*A*1 = *A*3	−0.64 (0.34)	−1.89(0.06)^#^	0.21(0.24)	0.88 (0.38)	0.42 (0.25)	1.64 (0.10)	−0.28 (0.26)	−1.07 (0.28)
*B*1 = *B*_3_	0.31 (0.16)	2.02 (0.04)^*^	0.06 (0.11)	0.53 (0.60)	−0.23 (0.12)	−1.95 (0.052)^#^	−0.06 (0.12)	−0.48 (0.63)
BIC full	2664.69 (4.10)		2504.28 (3.30)		2526.94 (2.94)		2529.81 (4.18)	
BIC linear	2653.95 (4.19)		2492.65 (3.31)		2514.44 (2.92)		2518.08 (4.16)	
**GRADE 6**								
*A*1	−0.28 (0.21)	−1.34 (0.18)	−0.26 (0.16)	−1.68 (0.09)	0.24 (0.15)	1.57 (0.12)	0.06 (0.17)	0.36 (0.72)
*B*1	0.12 (0.09)	1.32 (0.19)	0.15 (0.07)	2.07 (0.04)^*^	−0.15 (0.07)	−2.22 (0.03)^*^	−0.02 (0.08)	−0.30 (0.76)
*A*3	0.18 (0.21)	0.88 (0.38)	0.08 (0.16)	0.50 (0.62)	−0.09 (0.15)	−0.60 (0.55)	−0.04 (0.18)	−0.21 (0.83)
*B*3	−0.04 (0.09)	−0.43 (0.67)	−0.02 (0.07)	−0.25 (0.80)	0.04 (0.06)	0.64 (0.52)	−0.01 (0.07)	−0.12 (0.90)
*A*1 = *A*_3_	−0.46 (0.33)	−1.38 (0.17)	−0.34 (0.25)	−1.35 (0.18)	0.33 (0.24)	1.35 (0.18)	0.10 (0.28)	0.35 (0.73)
*B*1 = *B*_3_	0.16 (0.15)	1.08 (0.28)	0.17 (0.12)	1.46 (0.15)	−0.19 (0.11)	−1.76 (0.08)^#^	−0.01 (0.12)	−0.12 (0.91)
BIC full	2661.52 (2.15)		2506.37 (3.81)		2516.10 (1.85)		2532.63 (4.83)	
BIC linear	2650.25 (2.19)		2493.89 (3.80)		2503.98 (1.92)		2520.52 (4.86)	

Tabled values are from the four-parameter linear COMT X linear disorganization model in which intercept and slope values for the heterozygotes Met/Val group were constrained to be right in the middle between the corresponding values for the homozygous (i.e., Met/Met and Val/Val) groups. Tabled values are as follows: Estimate (SE) = point estimate of the parameter, with its associated standard error in parentheses; t (p) = t value for the point estimate, with the p-level of the t statistic in parentheses. The four rows for A_1_, B_1_, A_3_, and B_3_ refer to parameters in Equation 2 based on results from the linear X linear model (A_1_and B_1_, intercept and slope for the Met/Met homozygotes, respectively; A_3_and B_3_, intercept and slope for the Val/Val homozygotes, respectively. For the linear X linear model, the intercept and slope for Met/Val heterozygotes were the averaged intercept and slope between Met/Met and Val/Val). The row labeled A_1_ = A_3_ reports results of a test that A_1_ is equal to A_3_, consistent with the hypothesized model; and the row labeled B_1_ = B_3_ reports results of a test that B_1_ is equal to B_3_, consistent with the comparison model. The BIC values reported for the two alternative models are the mean BIC for the model, with SD of BIC values across the 100 imputation samples in parentheses.

# p < 0.10,

* p < 0.05.

In contrast to mother-reported data, it was never the case, no matter (a) how the *COMT* and disorganization predictors were parameterized (i.e., 2 or 3 levels) or (b) when the outcomes were measured (i.e., kindergarten or 6th grade), that teacher reports proved consistent with the anticipated effects for mother-reported child functioning.

Inspection of Figure 3 indicates that disorganized Met carriers (Met/Met, Val/Met) scored higher than disorganized Val/Val homozygotes on mother-reported positive behavior, even if not to a significant extent (Cohen's *d* = 0.439, 95% CI: [-0.109, 0.988]), with the reverse being true for mother-reported negative composites (Met carriers < Val/Val, *d* = −0.526, 95% CI: [-1.076, 0.025]). These results show the same patterns of difference in grade 6 ratings as shown in kindergarten ratings in Figure 2. When *COMT* was coded in 3-levels (i.e., Met/Met, Met/Val and Val/Val), the best-fitting linear gene model constrained intercept and slope estimates for the Met/Val heterozygotes to fall exactly midway between corresponding estimates for the homozygous groups, with results shown for mother ratings of positive and negative composites in kindergarten and grade 6 in Figures 4, 5, respectively. This model suggests that the gene effect may be linear, rather than nonlinear as in the original results by Hygen et al. (2014). That is, Hygen et al. and our analyses in Tables 2, 3 implicitly had a recessive gene effect specified, presuming that the presence of two Val alleles would lead to different outcomes than for the Met/Met and Val/Met groups, the latter of which would have identical outcomes.

The statistical foundation of the overall model fit for our sensitivity analyses are displayed in Tables 3, 4. In Table 3, statistical results are shown for grade 6 ratings by mothers and teachers using the same statistical models as for kindergarten ratings in Table 2. Inspection of results in Table 3 show that the grade 6 results are quite similar to the kindergarten results. The only exception is that the important test of *B*_1_ = *B*_2_ was significant at only a trend level (*p* < 0.10) on the mother-rated positive composite, although it remained significant (*p* < 0.05) for the mother-rated negative composite. As with the kindergarten ratings, all parameter estimates in Table 3 for the mother ratings were in the hypothesized direction, and none of the results for teacher ratings approached significance.

Turning to the results of the linear *COMT* X continuous disorganization analyses displayed in Table 4, findings for the best fit linear *COMT* X linear disorganization model are shown. Here, we reported parameter estimates (*A*_1_ and *B*_1_) for the Met/Met group and corresponding estimates (*A*_3_ and *B*_3_) for the Val/Val group; parameter estimates (*A*_2_ and *B*_2_) for the heterozygous Val/Met group were constrained to fall directly between corresponding values for the homozygous groups. None of the *A*_1_=*A*_3_ tests were statistically significant for mother-reported positive and negative behavior, revealing no differences between allelic groups for organized children. Results for the crucial *B* parameters were similar to, although not quite as strong, as for prior analyses. Specifically, the test of equality of *B* values was statistically significant for mother-rated positive composite in kindergarten (*p* < 0.05, R-square explained by the interaction term from the standard regression: mean = 0.008, *SD* = 0.002), but not in grade 6 (*p* = 0.28; R-square for the interaction term: mean= 0.002, *SD* = 0.0004). For the mother-rated negative composite, the difference in *B* values was significant at trend level in both kindergarten (*p* = 0.052; R-square for the interaction: mean = 0.007, *SD* = 0.002) and grade 6 (*p* = 0.08; R-square for the interaction: mean = 0.006, *SD* = 0.001). Still, all of the *B* values were in the hypothesized direction. Finally, no differences in either A or B parameters turned out significant in the case of teacher reports.

## Discussion

The purpose of the current investigation was to test the Norwegian hypothesis that the *COMT* genotype moderates the developmental legacy of disorganization vis-a-vis aggression and social competence during childhood in specific ways. Recall that Hygen et al. (2014) hypothesized that Val/Val homozygotes with histories of disorganization would exhibit more negative behavior as well as less positive behavior compared with their organized counterparts, seemingly reflecting a controlling-punitive style, whereas Met carriers would evince just the opposite pattern, such that those who were classified as disorganized would exhibit less negative and more positive behavior than non-disorganized/organized children, seemingly consistent with a controlling-caregiving style. Moreover, they predicted that such genetic moderation of attachment effects would not hold for children with organized attachment histories and would only prove evident when mothers characterized child behavior, not teachers. Recall, too, that the Norwegian investigators found support for their predictions only when looking at change over time in child functioning across the transition to school rather than at level of functioning prior to and following the school transition.

Here we sought to carry out a conceptual rather than exact replication of the original Norwegian hypothesis, focusing on the prediction from attachment at age 15 months to children's future functioning, first at the start of school and then 6 years later. In contrast to the Norwegians' reliance on a doll-play procedure administered at age four to measure disorganization, we employed the gold standard, Strange Situation at 15 months, relying on both the categorical and continuous measurement of disorganization. And, drawing on data from the NICHD Study of Early Child Care and Youth Development, we employed a confirmatory and competitive model-fitting approach that directly tested the anticipated and complex *patterning* of data predicted by the Norwegian hypothesis, while contrasting the hypothesized model with alternative ones (i.e., full and the comparison models). Of most importance, then, was not whether one or another component of the complex model was itself statistically significant, but whether the overall patterning of the data collectively proved consistent with predictions derived from the Norwegian hypothesis—and whether it did so when alternative parameterizations of disorganization and *COMT* were used.

Model-fit statistics, including those derived from robustness checks, like inspection of the graphs presented in the figures, indicated that Val/Val homozygotes with histories of disorganization in infancy exhibited—or tended to exhibit— more negative behavior as well as less positive behavior in kindergarten (as reported by mother) compared with their organized counterparts, seemingly reflecting a controlling-punitive style. In contrast, Met-carriers displayed—or tended to display—just the opposite pattern, such that those who were classified as disorganized in infancy exhibited less mother-rated negative and more positive behavior than non-disorganized/organized children, seemingly consistent with a controlling-caregiving style. Just as importantly, genetic-group differences did not emerge in the case of children with organized attachment histories, nor did any genetic-group differences emerge in the case of teacher reports of child behavior for either non-disorganized or disorganized children. Notably, this complex patterning of results emerged quite similarly regardless of whether *COMT* was coded as a 2-level variable (Met carrier vs. Val/Val) or 3-level variable (Met/Met vs. Met/Val vs. Val/Val), whether disorganization was coded categorically (D vs. not D) or continuously (using the D rating), and whether child behavior was predicted at kindergarten or Grade 6. To be noted, however, is that even with the large sample available for analysis, there were only a limited number of children with disorganized attachment histories. This resulted in even smaller subgroups of disorganized children when genotype was taken into consideration. In light of this limitation of small cell sizes, it seems notable that results proved strikingly similar when the continuous measure of disorganization was used in the secondary analyses.

The data from the both the primary and sensitivity analyses fit the Hygen et al. (2014) hypothesis that *COMT* would moderate the effect of attachment disorganization in a manner that could account for the divergent ways in which children manifesting disorganized attachments have been found to behave toward their mothers (controlling-caregiving vs. controlling-punitive). Having said that, it should be appreciated that the actual effect size of the COMT-X-disorganization interaction was small in magnitude at both kindergarten and 6th grade. Given that the interaction was anticipated and that GXE findings have proven challenging to replicate, even conceptually, we regard the results as meaningful. Nevertheless, what remains difficult to understand is why the targeted *COMT*-X-disorganization interaction only predicted change over time in the original Norwegian research and not levels of child behavior at either 4 or 6 years. Here, though, we found in our primary analyses that when disorganization was (a) measured categorically rather than continuously, (b) using the gold-standard Strange Situation rather than a story-stem completion task, and (c) at 15 months rather than at 4 years of age, that levels of kindergarten social behavior reported (only) by mothers could be accounted for by considering the moderation by *COMT* of attachment disorganization. Quite conceivably, these measurement and design differences across studies are responsible for the differences in findings across the two studies.

In any event, reasonably consistent support for the Norwegian hypothesis emerged in both inquiries as to why some children with disorganized attachment histories behave in seemingly controlling-caregiving and others in controlling-punitive ways. Especially notable, perhaps, is that in both studies the genetically-related divergent patterns of functioning are restricted to maternal reports of child behavior, thereby underscoring the relationship-specificity of disorganized attachment, at least when examined from the perspective of a gene -X-disorganization interaction. This could reflect the frightening experiences that children with disorganized attachment histories have had with their parents, but not with their teachers (e.g., Van Ijzendoorn et al., 1999; David and Lyons-Ruth, 2005). Alternative explanation of these relationship-specific findings) is that the detected gene -X-disorganization interaction might actually reflect passive gene -X-environment association. Recall, however, that this rGE possibility was discounted in the current study in that we (a) ruled out the association between Disorganization category (D vs. Not-D) and *COMT* genotype (Met carriers vs. Val/Val) and (b) detected no association between parent behavior (i.e., maternal sensitivity at both 6 and 15 months) and child *COMT* genotype (two levels and three levels) (see Supplemental Material, Table 1).

It is important to appreciate that our focus on *COMT* should not be read to imply that only this polymorphism may play a moderating role vis-à-vis the effects of disorganization. Rather, we focused exclusively on this candidate gene because we conceptualized this inquiry from its inception as an attempt to test an intriguing and most original hypothesis and, thereby, conceptually replicate and extend the work of Hygen et al. (2014). Moreover, it would be a mistake to presume that it is the action of *COMT per se* that accounts for the moderation detected, because *COMT* could function—statistically—as a moderator in this inquiry due to its association with some other gene that plays a truly functional role. Nevertheless, the fact that the *COMT* Val158Met gene regulates the degradation of dopamine in the PFC (Karoum et al., 1994; Lachman et al., 1996; Weinshilboum et al., 1999), thereby affecting dynamic dopamine metabolism in this brain region, provides biological plausibility as to why variation in *COMT* appears to account for variation in the functioning of children with histories of disorganized attachment, at least from the perspective of their mothers.

## Conclusion

Recent years have witnessed an outpouring of concern regarding the replicability of scientific findings (Jasny et al., 2011; Ryan, 2011). Perhaps nowhere has this issue emerged so forcefully in the human behavioral sciences as in research involving measured genes. Our current inquiry was motivated precisely by this concern for replication, which includes “conceptual replications” like that carried out here. That is, our replication of Hygen et al. (2014) did not involve repeating exactly what was done in their prior work; instead, it was directly *informed* by the Hygen et al. (2014) study in terms of structuring a research question, hypotheses, and analyses, albeit with certain variables that differed operationally, but not conceptually, from the Norwegian research. Indeed, we jumped at the opportunity to test the Norwegian hypothesis involving the interaction of one candidate gene, *COMT*, and disorganization once the initial test of it received some support. We regarded conceptual—as opposed to exact—replication as important because the real issue is not simply—or only—whether specific findings tied to specific measurements evaluated in a seemingly identical sample can be repeated, but whether the more general finding can be detected again using related, even if not exactly the same, measurements or design. Indeed, it is exactly this reasoning that undergirds virtually all meta-analyses.

It would, of course, be mistaken to claim that our conceptual replication of the Hygen et al. (2014) inquiry entirely resolves the issue of why some disorganized children develop in one way—or at least develop certain kinds of relationships with their mothers which take one form—rather than another. Indeed, the fact that variation in *COMT* accounts for some variation in how disorganized infants develop should not be read to imply that other factors, including perhaps especially, parental behavior, are not important. We would strongly encourage investigators to adopt, as we have endeavored to, statistical techniques that directly fit the statistical glove to the theoretical/conceptual hand rather than opting for more traditional exploratory approaches.

## Author contributions

ZL was involved in statistical analyses, result interpretation and drafting the manuscript. BH participated in the study design and critical revision of the manuscript. KW was involved in designing the confirmatory model-testing framework, statistical analyses, interpreting results, and drafting the manuscript. TB participated in the study design and critical revision of the manuscript. LW participated in the study design and critical revision of the manuscript. JB participated in conceiving the study, interpreting results and helped to draft the manuscript. All authors read and approved the final manuscript, and agree to be accountable for all aspects of the work in ensuring that questions related to the accuracy or integrity of any part of the work are appropriately investigated and resolved.

## Funding

This research was funded by grants 191144/V50 and 228685/H10 from the Research Council of Norway to Dr. Wichstrøm.

### Conflict of interest statement

The authors declare that the research was conducted in the absence of any commercial or financial relationships that could be construed as a potential conflict of interest.
