# Supplementary material for: Disorganization, COMT, and Children's Social Behavior: The Norwegian Hypothesis of Legacy of Disorganized Attachment
Source: Front Psychol. 2016 Jul 12;7:1013. doi: 10.3389/fpsyg.2016.01013 (PMC4940399; doi:10.3389/fpsyg.2016.01013)
Supplement: Supplementary file 1 [file Table1.docx]

Supplementary Material

Disorganization, *COMT*, and Children’s Social Behavior:

The Norwegian Hypothesis of Legacy of Disorganized Attachment

Zhi Li^*^, Beate Wold Hygen, Keith Widaman, Turid Suzanne Berg-Nielsen, Lars Wichstrøm, Jay Belsky

*** Correspondence:** Zhi Li: zhdli@ucdavis.edu

**Supplementary Table 1**.
Testing association between child COMT genotype (2-level and 3-level coding) and maternal sensitivity at child age six months and 15 months.

|  | Maternal sensitivity@ 6month | Maternal sensitivity @15month |
| --- | --- | --- |
| COMT  (Met carrier vs. Val/Val) | Independence T-test indicated no significant difference in 6-month maternal sensitivity for Met carriers (Mean= 9.54, *SD*= 1.69) and Val/Val (Mean= 9.64, *SD*= 1.59), *t* (550) = -0.67, *p*= 0.50.  *N*(Met carriers)= 401, *N*(Val/Val)= 151 | Independence T-test (unequal variances, *F*= 4.66, *p* = 0.03) indicated no significant difference in 15-month maternal sensitivity for Met carriers (Mean= 9.79*, SD*= 1.40) and Val/Val (Mean= 9.56, *SD* = 1.58), *t* (242. 35)= 1.54, *p* = 0.12.  *N* (Met carriers) = 407,  *N*(Val/Val)= 151 |
| COMT  (Met/Met, Val/Met, Val/Val) | One-way ANOVA indicated no significant effect of COMT (Met/Met, Val/Met, Val/Val) on 6-month maternal sensitivity, *F* (2, 549) = 0.25, *p* = 0.78.  N(Met/Met) = 145, N(Met/Val) = 256,  N(Val/Val)= 151 | One-way ANOVA indicated no significant effect on COMT (Met/Met, Val/Met, Val/Val) on 15-month maternal sensitivity, *F* (2, 555) = 1.84, *p* = 0.16.  N(Met/Met) = 146, N(Met/Val) = 261, N(Val/Val) = 151. |
